# Importance of TREC and KREC as molecular markers for immunological evaluation of down syndrome children

**DOI:** 10.1038/s41598-023-42370-0

**Published:** 2023-09-18

**Authors:** Eman Eissa, Hanan H. Afifi, Assem M. Abo-Shanab, Manal M. Thomas, Mohamed B. Taher, Rania Kandil, Naglaa M. Kholoussi

**Affiliations:** 1 Department of Immunogenetics, Human Genetics and Genome Research Institute, National Research Centre, Cairo, Egypt; 2 Department of Clinical Genetics, Developmental Assessment and Genetic Disorders Clinic, Human Genetics and Genome Research Institute, National Research Centre, Cairo, Egypt

**Keywords:** Immunology, Biomarkers, Diseases

## Abstract

Recurrent and severe infections occurred in children with Down Syndrome (DS) due to immunological parameter defects have been reported. The aim of the study is to evaluate the importance of using T-cell receptor excision circle (TREC) and kappa-deleting recombination excision circle (KREC) as molecular markers for immunological investigation of children with DS. The study included 40 non-disjunction trisomy 21 confirmed DS children, and 25 healthy controls. Peripheral blood (PB) was analyzed for lymphocyte subpopulations by flow cytometry, serum immunoglobulin levels, and TREC and KREC copy numbers using quantitative real-time PCR. DS patients showed significantly lower absolute counts of PB T lymphocytes, T helper lymphocytes, T cytotoxic lymphocytes, B lymphocytes, and Natural killer cells, and lower serum IgA, IgG, and IgM levels compared to healthy controls. Copy number of TREC and KREC showed no significant differences between DS patients and healthy controls. There is a significant positive correlation between TREC copy number with a percentage and absolute count of helper T lymphocytes in patients. Also, the KREC copy number was significantly negatively correlated with the age of patients. These findings suggest that copy numbers of TREC and KREC could be useful as molecular markers for immunological evaluation of patients with DS.

## Introduction

Down syndrome (DS), trisomy 21, is a common cause of mental sub-normality and is the most common chromosomal abnormality. The frequency of DS is approximately 1:770 births, with a little male predominance^1^. More than 40 features that may be associated with DS as craniofacial abnormalities, hypotonia, heart defects, duodenal atresia, mental retardation, and dermatoglyphics. Nevertheless, not all features are observed in one individual with DS, but variable features occur to some degree in each individual with trisomy 21, the mechanism for phenotypic variability is not understood^2^. There are various medical features associate with DS children including short stature, mental retardation, umbilical hernia, etc.^3^.

Recurrent and severe infections have been reported in children with DS mostly respiratory infections^4^. This may be due to overexpression of chromosome 21 encoded gene products which impairs the immune response in people with DS^5^. Individuals with DS may have an abnormal immune system as well as a defective lymphocyte count. They could exhibit deficient T lymphocyte response and reduction of immunoglobulins^6^. However, differences in numerous immune response compartments have been reported. Various subsets of lymphocytes have been discovered to be significantly diminished in youth, and elevated with aging. T and B cell subsets showed reduced levels below 10% of normal in nearly 90% of DS children and below 5% in 60% of them. Early T lymphocyte growth in infancy was not normal^7^.

Anatomic evidences show that immunological insufficiency in DS patients is caused mostly by thymus malfunction, which results from lymphocyte depletion, cortical reduction, loss of corticomedullary delimitation, and thymic medulla hypertrophy of Hassall’s corpuscles. As a result, thymic maturation is aberrant, resulting in phenotypic and functional defects in circulating T lymphocytes^8^.

Serum levels of immunoglobulins may vary according to the age in DS children. It has been found that the absolute number of IgG and IgA antibodies rises in children after the age of six and IgM levels slightly decreases in adolescence^9^. Moreover, there is a low number of circulating B lymphocytes, T lymphocytes and Natural Killer cells in those children^10–12^. Also, functions of T and B lymphocytes have been analyzed in DS. In DS, the proliferative response of lymphocytes to phytohemagglutinin was considerably lower. Not all DS patients have disrupted levels of immunoglobulins, only some DS children exhibit lower IgG levels for age^13–15^.

During normal development and maturation of T lymphocytes in the thymus, recombination of T lymphocyte receptor gene occurs causing its splice and rearrangement. In this process, DNA segments are excised from T cell receptor (TCR) gene and circularized forming T cell receptor excision circle (TREC). Number of TREC produced can reflect the number of naive T lymphocytes passed through the thymic maturation process and thus the activity of thymus^7,16–19^. Similarly, kappa-deleting recombination excision circle (KREC) are produced during B lymphocyte development as circularized DNA fragments excised from B cell immunoglobulin kappa gene^17–19^.

TREC and KREC have been used to assess the activity of the immune system in newborn screening for severe combined immunodeficiency disorder (SCID). It has been reported that TREC and KREC are T and B cell development indicators, respectively. They are effective tools for assessing T and B cell activity and immunological reconstitution^20–22^.

In this study, we intended to evaluate the immunological profile of DS patients by elucidating the importance of using TREC and KREC as molecular markers for immunological investigation. We investigated percentages and absolute count of peripheral blood (PB) lymphocyte subsets and serum levels of immunoglobulins. Also, we determined copy numbers of TREC and KREC in PB and evaluated their associations with the immunological parameters and the clinical manifestations of DS patients.

## Results

### Clinical data

The study included 40 DS patients aged 6 months to 7 years old. They were 24 males (60%) and 16 females (40%). The median age (Interquartile range (IQR)) at the time of sampling was 1.8 (2) years. Their chromosomal analysis revealed non-disjunction trisomy 21 in all cases. Associated clinical abnormalities e.g. hypothyroidism, mild congenital cardiac anomalies (PFO or ASD), and umbilical hernia were present in some patients (Table 1).

**Table 1:** Demographic, clinical data, and hematological findings of the study participants.

Demographic data	N (%)	
	Down syndrome patients	Healthy controls
Gender (male/female)	24/16 (60/40)	9/16 (36/64)
Age, median (IQR), years Range	1.8 (2) (0.5–7)	3 (2.5) (1–6)
Parental consanguinity	7 (17.5)	–
Parental residence (Rural/Urban)	19/21 (47.5/52.5)	–
Father age at birth (mean ± SD), years Range	39.2 ± 6.8 (24–59)	–
Mother age at birth (mean ± SD), years Range	34.6 ± 6.6 (16–46)	–
**Associated clinical manifestations**		
Hypothyroidism	5 (12.5)	–
Congenital heart anomalies (PFO or ASD)	18 (45)	–
Umbilical hernia	5 (12.5)	–
No associated anomalies	12 (30)	25 (100)
**Hematological findings**		
Hb (g/dl) (mean ± SD) Range	11.9 ± 0.9 (9.5–13.9)	11.5 ± 1.5 (9.3–16.2)
Total Leucocytic count (10^3^/μl) (mean ± SD) Range	6.7 ± 2 (3.5–12.9)	6.9 ± 2.2 (4.1–10.9)
Lymphocyte count (10^3^/μl) (mean ± SD) Range	3.3 ± 1.2 (1.7–6.4)	3.9 ± 1.8 (1.3–9.1)
Lymphocyte % (mean ± SD) Range	50.2 ± 12 (24–79.2)	56 ± 17.4 (23–87.5)

*IQR, Interquartile range; SD, Standard deviation; PFO, Patent foramen ovale; ASD, Atrial septal defect.

### Immunological profile of DS patients

**Figure 1:**
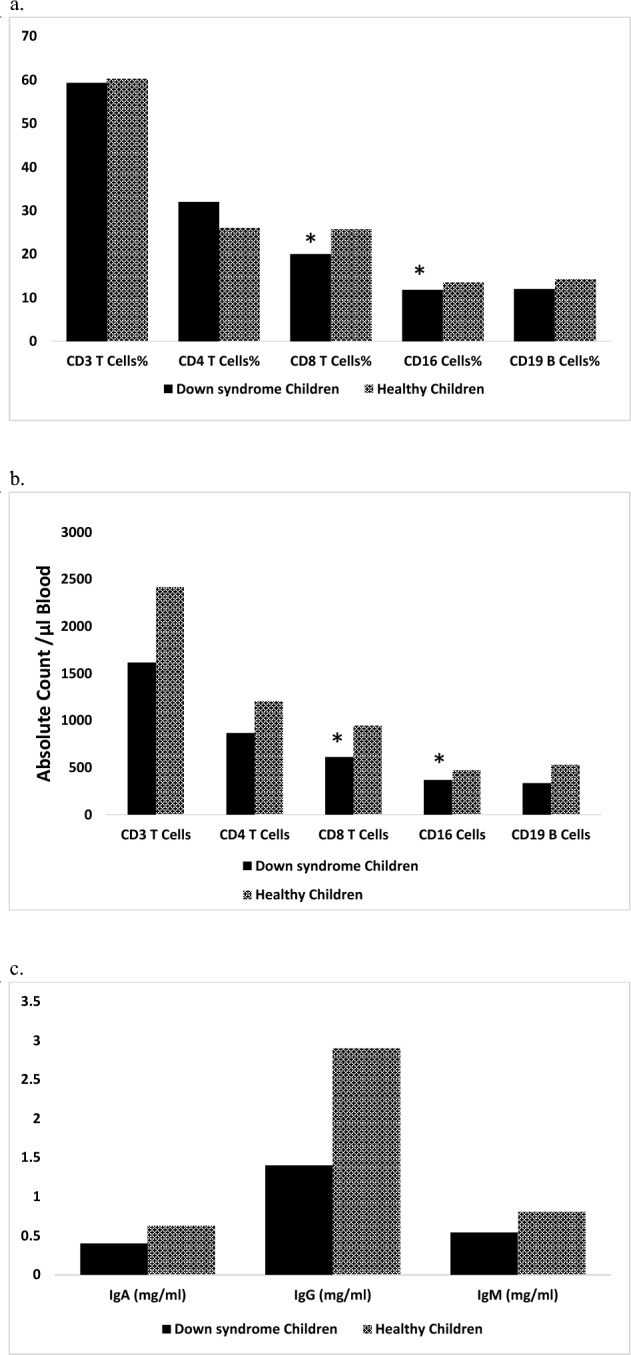
Immunological parameters of Down syndrome patients compared to healthy controls. Data were expressed as a median. *Statistically significant at *P* < 0.05 (by Mann–Whitney U test).

Our results have indicated that percentages of T lymphocytes, T helper lymphocytes, T cytotoxic lymphocytes, B lymphocytes, and Natural killer cells in PB of DS patients are comparable to those of healthy controls (Fig. 1a) while their absolute counts are lower than those of healthy controls (Fig. 1b). DS patients have lower immunoglobulin levels (IgA, IgG, and IgM) in serum compared to that of controls (Fig. 1c).

### Association of Immunological profile with the clinical manifestations of DS patients

**Figure 2:**
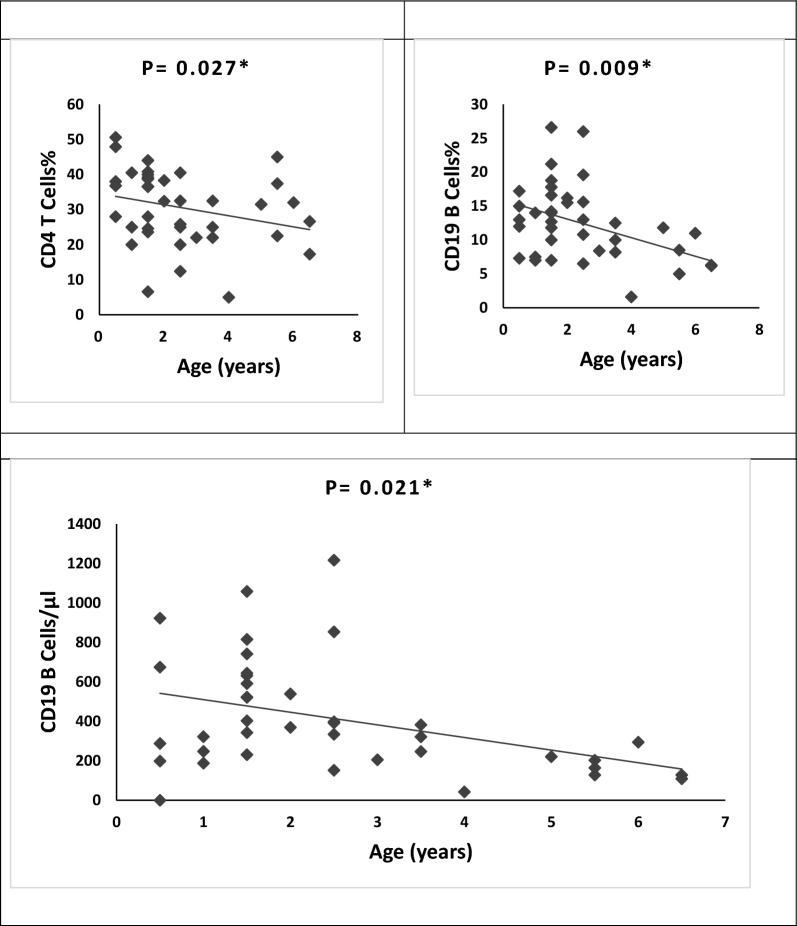
Association of Immunological profile with the age of Down syndrome patients. *Statistically significant at *P* < 0.05 (by Spearman correlation analysis).

Our association analysis showed that the percentage and absolute count of B lymphocytes and percentage of helper T cells have significant negative associations with the age of DS patients (Fig. 2). However, there is no association between the immunological parameters of patients and their associated clinical manifestations e.g. hypothyroidism, congenital heart anomalies, and umbilical hernia. Also, there is no association between the immunological parameters of patients and their parental consanguinity, residence (rural/urban), or associated clinical manifestations.

### TREC and KREC copy numbers and their correlation with the immunological parameters and clinical characteristics of DS patients

**Figure 3:**
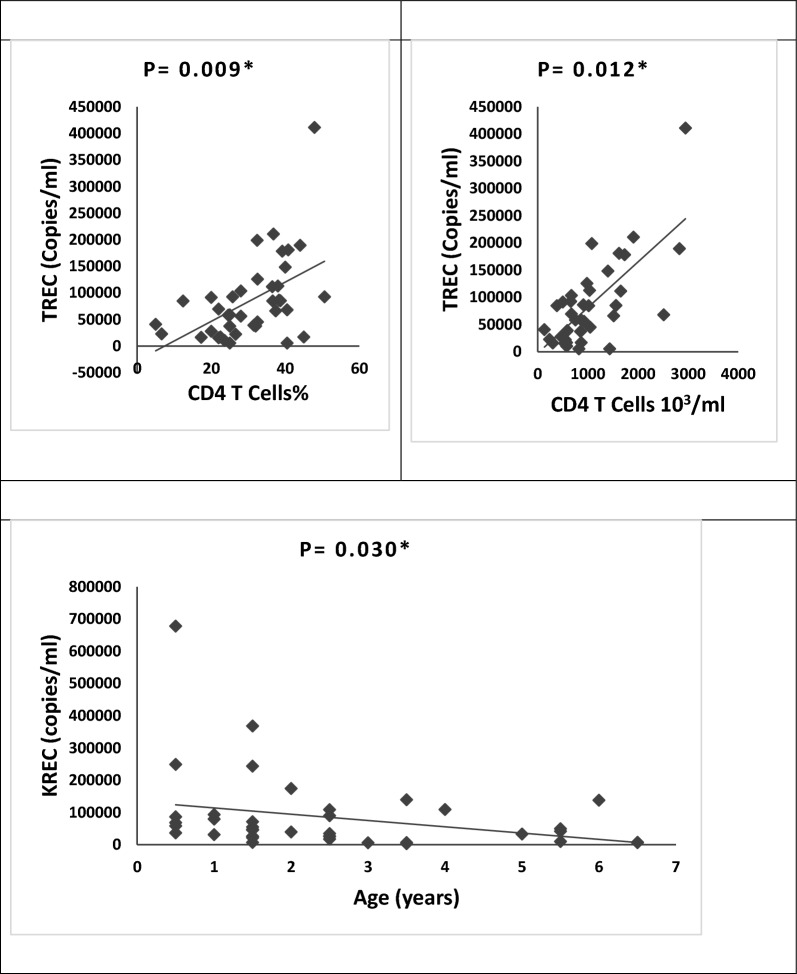
Correlation of TREC and KREC copy numbers with the immunological parameters and age of Down syndrome patients. *Statistically significant at *P* < 0.05 (by Spearman correlation analysis).

Copy number of TREC and KREC showed no significant differences between patients and healthy controls. There is a significant positive correlation between TREC copy number with a percentage and absolute count of helper T cells in patients. Also, the KREC copy number was significantly negatively correlated with the age of patients (Fig. 3). Otherwise, no associations were found between TREC and KREC copy numbers and the associated clinical manifestations of patients, parental consanguinity, or patients’ residence (rural or urban).

### The receiver operating characteristic (ROC) curve of TREC and KREC and their cut-off values

**Figure 4:**
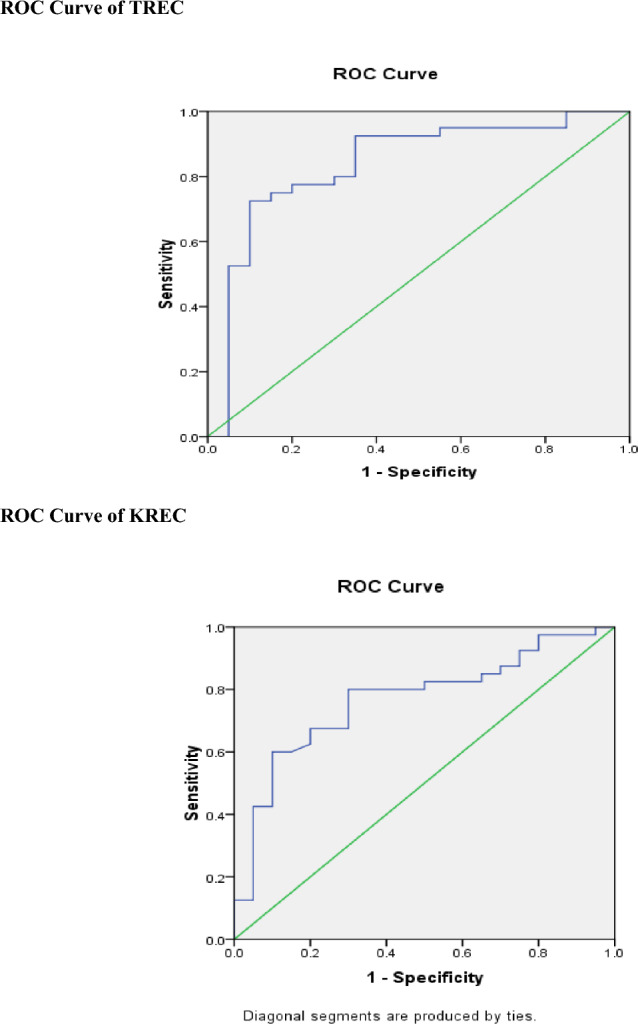
ROC curve of TREC and KREC.

The ROC curve of TREC showed a good significant area under the curve (AUC) value of 0.837 with a sensitivity of 93% and specificity of 65% (*P* < 0.001); while KREC showed a ROC curve with a significant AUC value of 0.768, sensitivity 80% and specificity 70% (*P* ≤ 0.001) (Fig. 4, Table 2).

**Table 2:** AUC, sensitivity, specificity, and Cut-off values of TREC and KREC.

	AUC	Standard error**	95% Confidence Intervals	Sensitivity (%)	Specificity (%)	Cut-off value
TREC	0.837*	**0.060**	**(0.720–0.955)**	**93**	**65**	**15,270**
KREC	0.768*	**0.064**	**(0.644–0.893)**	**80**	**70**	**19,887**

*Statistically significant at *p* ≤ 0.001 versus controls.

**Under the nonparametric assumption.

Significance values are [bold].

## Discussion

The results presented in this study provide insights into the immunological profile of DS patients and their potential associations with clinical manifestations. The baseline characteristics of the study participants showed a predominantly male population with a median age of 1.8 years. The cumulative clinical manifestations of patients included thyroid, cardiac, and hernia disorders. The immunological profile analysis have revealed that the percentages of T lymphocytes, T helper lymphocytes, T cytotoxic lymphocytes, B lymphocytes, and natural killer cells in PB of DS patients are comparable to those of healthy controls, while their absolute counts are lower in patients than those of controls. Moreover, DS patients have lower immunoglobulin levels (IgA, IgG, and IgM) in serum compared to controls. Our findings are consistent with previous studies on the immune system in individuals with DS. Ram and Chinen^7^ and Huggard et al.^23^ have demonstrated that the lower absolute counts of immune cells and immunoglobulin levels observed in individuals with DS may contribute to an increased susceptibility to infections and a reduced ability to mount an effective immune response. Also, Dieudonné et al.^24^ have reported immune dysfunction in DS patients. Our association analysis showed that the percentage and absolute count of B lymphocytes and percentage of helper T cells have significant negative associations with the age of patients. This is in agreement with a study by Froňková et al.^18^ who demonstrated a reduction of absolute B and T lymphocyte counts with aging. However, there are no significant associations between the immunological parameters of our DS patients and their associated hypothyroidism, congenital heart anomalies, or umbilical hernia.

This study has found that there are no significant differences in TREC and KREC copy numbers between DS patients and healthy controls. Moreover, no associations have been found between TREC and KREC copy numbers and the clinical characteristics of DS patients. In contrast, other studies have found that the copy numbers of TREC and KREC were significantly lower in DS patients compared to healthy controls, suggesting a decrease in the output of newly produced T and B cells in DS patients which may contribute to immune defects and increased susceptibility to infections observed in individuals with DS^25–27^.

Interestingly, our study has found a significant positive correlation between TREC copy number and the percentage and absolute count of CD4 T lymphocytes in DS patients indicating a relationship between the molecular markers and the respective lymphocyte subsets. Similarly, a study by McCullough et al.^28^ showed a positive association between TREC and the percentage of CD4 and CD8 T lymphocytes, recommending that TREC may be a useful biomarker for monitoring immunological insufficiency in DS patients. Additionally, we observed a significant negative correlation between copy numbers of KREC and age in DS patients, which is consistent with previous studies which reported age-related declines in thymic and bone marrow outputs^18,29,30^, suggesting that KREC could be a helpful biomarker for monitoring age-related immune defects in DS patients.

Furthermore, we have performed ROC curve analysis to assess the diagnostic accuracy of TREC and KREC copy numbers as molecular markers for immune insufficiency in DS patients. Our findings showed good significant AUC values, with TREC having a sensitivity of 93% and specificity of 65%, and KREC having a sensitivity of 80% and specificity of 70%. ROC curve analysis demonstrated that TREC and KREC have high sensitivity and specificity for detecting immune defects in DS patients, indicating that these biomarkers have potential diagnostic value for immune dysregulation in DS. This matched with other studies that found that TREC and KREC had high sensitivity and specificity for detecting immune deficiency, supporting their potential utility as molecular markers for immunological investigations in DS population^19,31^.

The main limitation of our study included the small sample size, which may limit the detection of significant associations between TREC/KREC and clinical data of DS patients. Therefore, further researches on a large sample size are needed in the future to investigate the clinical associations with TREC/KREC in DS patients.

## Conclusion

Our study provides evidence of immune defects in DS patients, as well as the potential utility of TREC and KREC as molecular markers for immunological evaluation of those patients. The findings also highlight the importance of considering age-related changes in the immunological profile of those patients when interpreting immunological data. However, there is a need for large-scale studies to confirm these findings.

## Methods

### Ethics approval and consent to participate

This study was approved by the ethics committee of the National Research Centre, Egypt with an approval number 19267 and has therefore been performed under the ethical standards laid down in the 1964 Declaration of Helsinki and its later amendments. All samples were obtained with the written informed consent of the parent/guardian of all children involved in this study before their enrollment.

### Study participants

This study included 40 DS patients and 25 healthy subjects matched for age and sex (age range: 6 months to 7 years). They were recruited from the Developmental Assessment and Genetic Disorders Clinic, National Research Centre, Egypt. Enrolled children were subjected to history taking, pedigree analysis, and meticulous clinical examinations. G-banding chromosomal analysis was performed on all patients to confirm the diagnosis of trisomy 21. A thyroid profile, electrocardiogram (ECG), and abdominal sonar were done for all DS patients to exclude or confirm any associated anomalies. Demographic and cumulative clinical manifestations were recorded. Subjects with missing values were excluded during collecting data before beginning of the study. Follow-up observation time was one year.

### Laboratory methods

#### Flow cytometry analysis

PB samples were collected from patients and healthy controls in tubes containing EDTA.50 μL of EDTA-treated PB were incubated for 30 min at 4 °C in the dark with fluorochrome-labeled monoclonal antibodies (mAbs): Fluorescein Isothiocyanate (FITC)-conjugated CD3, Allophycocyanin (APC)-conjugated CD19, Phycoerythrin (PE)-conjugated CD4, FITC-conjugated CD8, and PE-conjugated CD16 (Becton Dickinson, USA). Red blood cells were lysed using BD FACS Lysing Solution (Becton Dickinson, USA). The stained cells were then washed and resuspended in phosphate-buffered saline. Approximately 30,000 stained cells in each sample were analyzed with a BD Accuri™ C6 Flow Cytometer (BD Biosciences). The lymphocytes were gated by setting the appropriate forward scatter/side scatter axes. Data were acquired and data analysis was performed by the BD Accuri™ C6 software program^32^.

#### Determination of serum immunoglobulin levels

Serum immunoglobulin levels (IgA, IgG, and IgM) were determined in patients and healthy controls using Human ELISA kits (SunRed Biotechnology Company, Shanghai, China) according to the manufacturer’s instructions.

#### DNA isolation

DNA was isolated from 200 µl of PB using QIAamp DNA Blood Mini Kit (Qiagen GmbH, Hilden, Germany) by following the manufacturer’s instructions.

#### TREC and KREC quantification

Copy numbers of TREC and KREC were determined using Quantitative Real-Time PCR assay performed on the 7500 Fast Real-Time PCR (Applied Biosystems). Primers and probes of TREC, KREC, and the reference gene TRAC were designed according to Sottini et al. (2010). TaqMan Universal master mix (Applied Biosystems) was used in the reaction. The protocol of reaction was as follows: 50 °C for 2 min, 95 °C for 10 min, followed by 45 cycles at 95 °C for 15 s and 60 °C for 1 min. TREC, KREC, and TRAC copy numbers were obtained by deducing the respective sample quantities from the standard curve obtained by serial dilutions (10^6^, 10^5^, 10^4^, 10^3^, 10^2^, and 10) of the linearized triple-insert plasmid DNA (obtained from Alessandra Sottini and Luisa Imberti, Italy) that amplified in each PCR plate^33^.

### Statistical analysis

Data were statistically analyzed using SPSS version 27.0 software (SPSS Inc., Chicago, Illinois, USA). The Non-parametric Mann–Whitney U test was used for comparing the immunological parameters and immunoglobulin levels between groups of the study. Associations among the immunological parameters, immunoglobulin levels, TRECs and KRECs, age, and the clinical manifestations of patients were analyzed using Spearman’s rank correlation. Data were presented as mean ± SD or median (IQR). A *P*-value of less than 0.05 was considered statistically significant. ROC curve was constructed for TREC and KREC to evaluate the efficiency of those markers for immunological investigation in DS patients. AUC values, specificity, sensitivity, and 95% confidence intervals were calculated^34^.

## Data Availability

The datasets used and/or analyzed during the current study are available from the corresponding author on reasonable request.
